# Role of surgical embolectomy in the management of acute massive and submassive pulmonary embolism in the setting of a small island developing state

**DOI:** 10.1093/jscr/rjad468

**Published:** 2023-08-16

**Authors:** Ninon Forter-Chee-A-Tow, Alan Smith

**Affiliations:** The Queen Elizabeth Hospital, Bridgetown, St Michael, Barbados; University of the West Indies at Cave Hill, Faculty of Medical Sciences, The Queen Elizabeth Hospital, Bridgetown, St Michael, Barbados

**Keywords:** Acute pulmonary embolism, surgical embolectomy, index case

## Abstract

Acute pulmonary embolism (PE) remains a life-threatening condition despite advances in diagnostic and therapeutic modalities. Treatment modalities include systemic thrombolysis, catheter-based therapies and surgical embolectomy. This case report describes the first recorded surgical embolectomy for acute PE in Barbados, a small island developing state.

## INTRODUCTION

Acute pulmonary embolism (PE) remains a life-threatening condition despite advances in diagnostic and therapeutic modalities with an estimated incidence rate of 39–115 per 100 000 population as reported by the European Society of Cardiology. Increasing awareness and access to diagnostic modalities have increased clinical suspicion and confirmation of PE. Risk classification aids in decision of therapeutic management, need for hospitalization and mortality. While treatment modalities continue to evolve, surgical embolectomy has remained an underutilized tool in the armamentarium for treatment. In this case report we present the first reported successful pulmonary embolectomy for treatment of acute PE in Barbados.

## CASE REPORT

A 50-year-old female with no known chronic illnesses presented 2 weeks after total abdominal hysterectomy and bilateral salpingo-oophorectomy for symptomatic uterine fibroids. On presentation, she complained of a 6-day history of shortness of breath with palpitations, was ambulant postoperatively with no anticoagulation on discharge and review of her medical file noted no use of pneumatic devices in the perioperative period. Presentation was because of a 1-day history of worsening symptoms now occurring at rest.

**Figure 1:**
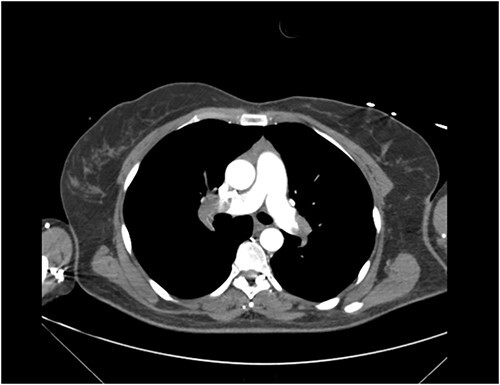
CT Pulmonary Angiogram demonstrating filling defects in the left and right pulmonary arteries.

Clinical examination revealed a middle-aged female in respiratory distress. Vitals on triage were blood pressure of 146/103 mmHg, pulse rate of 110 beats per minute, respiratory rate of 31 beats per minute and saturation of 67% on oxygen at 15 L/min. Well’s score was calculated at 6.0. CT pulmonary angiogram revealed extensive PE with filling defects in the right and left pulmonary arteries extending into the right lobar and segmental arteries, left upper segmental and lingula branches (Fig. 1). A bedside echocardiogram revealed evidence of right heart strain.

**Figure 2:**
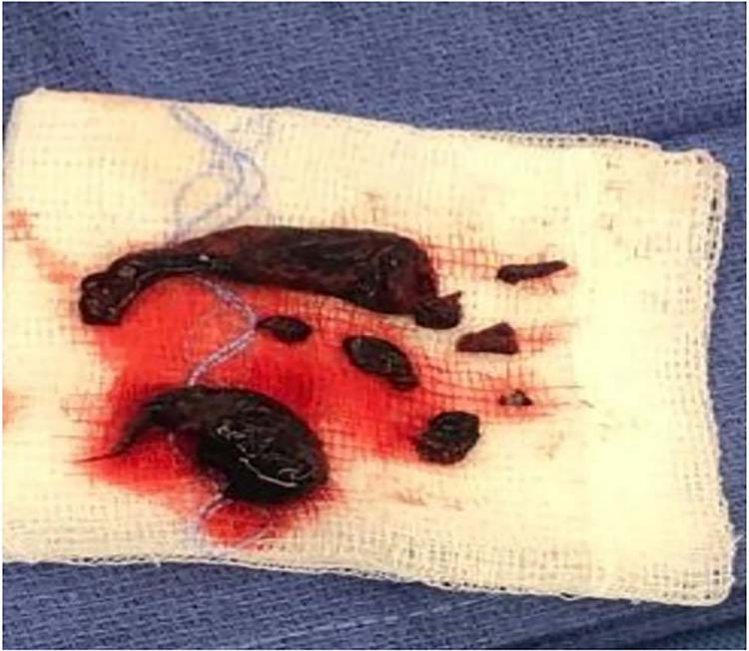
Extracted emboli from the index case.

The patient was commenced on anticoagulation and intubated in the emergency department because of worsening oxygenation despite trial of noninvasive ventilation. Given her clinical status and investigations, the decision was made to perform an emergency surgical pulmonary embolectomy after discussion with her managing teams and family. Pressor support was commenced upon transfer to the operating room because of persistent hypotension. Surgical embolectomy was performed via median sternotomy, and use of cardiopulmonary bypass with no aortic cross-clamping or cardioplegic cardiac arrest. Removal of clot was performed via incisions in both right and left pulmonary arteries with extraction using suction and thoracoscopic forceps (Fig. 2). Postoperative course was complicated by a ventilator-associated pneumonia. An official echocardiogram performed postoperatively revealed an ejection fraction of 50%, mild global hypokinesis, a severely enlarged right atrium and mild/moderate tricuspid regurgitation. The right ventricle was enlarged with preserved systolic function and pulmonary artery systolic pressure of 50 mmHg. Pulmonology referral was made, and Sildenafil commenced because of nil availability of Riociguat. Six months post-event the patient reported ambulating half a mile without symptoms and was awaiting repeat echocardiogram. She had no limitations, shortness of breath and was able to recommence gym training.

## DISCUSSION

Historically, surgical embolectomy was associated with poor outcomes and high early mortality (>30%) dating back to its inception in the 1960s. Until the early 2000s, this procedure was reserved for patients in extremis. In early 2000s, increasing access to imaging and reports demonstrating benefits of early surgical intervention renewed interest in this treatment modality.

Indications for surgical management in the setting of massive acute PE include contraindications to thrombolysis, failed thrombolysis, free-floating thrombus and concomitant cardiac pathology. Persons with contraindications to thrombolysis include but are not limited to recent brain/spinal, major surgery including pelvic surgery, stroke within the last 3 months and bleeding diathesis. The index case presented post-recent major pelvic surgery and was not a candidate for systemic thrombolysis. A study by Meneveau *et al*. [1] showed lower mortality rates for persons who underwent surgical embolectomy post-failed thrombolysis versus persons with repeat attempt at thrombolysis (7 versus 38%, respectively). The presence of free-floating thrombus in the right heart has been associated with higher early mortality reported as ~80% [2]. Concomitant cardiac pathology such as a patent foramen ovale is also considered an indication for surgical management [2].

Indications in the setting of submassive PE are similar to those for massive PE with additionally, the presence of moderate–severe right ventricular (RV) dysfunction (intermediate-high risk). Presence of right heart dysfunction is correlated with worse outcome and a 3-fold increased risk of death within 30 days [1].

Absolute contraindications to surgical management include acute on chronic PE (because of the high risk of persistent pulmonary hypertension, right heart failure and intractable pulmonary hemorrhage), active bleeding (because of risk of potential hemorrhage) and lack of qualified personnel, surgical equipment and ICU facility. Surgical embolectomy has demonstrated improvements in mortality and quality of life for persons presenting with acute PE. A study by Lee *et al*. [3] aimed to determine survival and recurrence rates between patients who underwent thrombolysis versus surgical embolectomy as first-line therapy with overall, no significant difference demonstrated between the types of reperfusion treatment regarding 30-day mortality (15 and 13%, respectively). However, thrombolysis was associated with a higher risk of stroke and re-intervention at 30 days. Thrombolytic therapy was also associated with a higher rate of recurrent PE requiring readmission (7.9 versus 2.8%) [3].

Major limitations in offering surgical reperfusion within our setting include timely diagnosis and intervention as well as lack of access to extracorporeal membranous oxygenation (ECMO) and ventricular assist devices. ECMO and ventricular assist devices have been used as a bridge to thrombolysis or surgical management particularly in persons presenting with massive PE [4]. In addition, cardiopulmonary support allows for improvement of RV function and restoration of systemic blood pressure and RV coronary perfusion. Meneveau *et al*. [1] reported significant improvement in outcomes with combination of ECMO and surgical management in persons with massive PE requiring cardiopulmonary resuscitation. Additional limitations within our setting include limited access to blood products and resources including surgical equipment.
